# A bibliometric study of global trends in diabetic nephropathy and intestinal flora research

**DOI:** 10.3389/fmicb.2025.1577703

**Published:** 2025-05-21

**Authors:** Nuo Wang, Xinyu Li, Hanjun Weng, Yijun Zhang, Qiurui Li, Xiaoxiao Luo, Yu Chen, Yiyang Dong

**Affiliations:** Medical School of Shihezi University, Shihezi, Xinjiang, China

**Keywords:** diabetic nephropathy, gut microbiome, bibliometrics, CiteSpace, VOSviewers

## Abstract

**Background:**

Diabetic nephropathy is chronic kidney damage caused by diabetes and is one of the most common microvascular complications of diabetes. In diabetic patients, prolonged hyperglycemia leads to progressive damage to kidney structure and function. With the increasing incidence of diabetes, the number of patients with Diabetic Nephropathy is also increasing year by year. At present, there is no drug to cure Diabetic Nephropathy. More and more evidence shows that the development of Diabetic Nephropathy is inseparable from the intestinal axis, and the disorder of intestinal flora is related to the progress of diabetes. Maybe we can explore the pathogenesis of Diabetic Nephropathy from the intestinal flora and find new methods to treat Diabetic Nephropathy.

**Methods:**

This article uses CiteSpace VOSviewer and Bibliometrix statistical software explore research hotspots and trends of intestinal flora and Diabetic Nephropathy. The Web of Science Core Collection (WoSCC) was searched for literature from database establishment to December 4, 2024, and ultimately 238 articles were included for quantitative analysis.

**Results:**

The number of publications has been increasing year by year, reaching its peak in 2024. The high-yield institution is Beijing University of Chinese Medicine, and the most productive country is China. Zhang Yi ranks first in the number of publications by the author. After removing the theme word, inflammation appears the most frequently, followed by oxidative stress. The outbreak hotspots are mainly concentrated in uremic toxin, short chain fatty acid, soy milk, aryl hydrocarbon receptor.

**Conclusion:**

The exploration of the mechanism of action and therapeutic or adjuvant therapeutic targets of the gut microbiome and its metabolites in DN patients may become a research hotspot in the future direction of DN and gut microbiome. Inflammation, oxidative stress, and the production of urinary toxins in DN patients are the directions for researchers to explore the mechanisms related to DN patients and gut microbiome. Aryl hydrocarbon receptor (AhR), Short-chain fatty acids (SCFAs), Traditional Chinese medicine and soy milk provide researchers with treatment ideas for diabetic nephropathy. Exploring the specific mechanisms and therapeutic effects of DN and gut microbiome requires cohort studies and clinical trials for validation.

## 1 Introduction

According to the data released by the International diabetes Federation (IDF) in 2021, about 537 million people worldwide have diabetes (Saeedi et al., 2019). Among them, about 40% of patients will develop Diabetic Nephropathy (DN), and about 75% of DN will become end-stage nephrosis (Du et al., 2021). Once it progresses to end-stage renal disease, renal replacement therapy is needed, making DN an increasingly growing public health issue and a global economic burden (Mosterd et al., 2021). The treatment drugs for DN include angiotensin blockers and novel hypoglycemic drugs, but these cannot prevent DN from further progressing to renal failure, nor can they completely treat DN (Fan K. et al., 2023). Therefore, there is an urgent need to find new treatment methods and drugs.

There are a large number of microorganisms in the gastrointestinal tract called gut microbiome, and in recent years, more and more evidence has shown that the disorder of gut microbiome is related to the progression of diabetic nephropathy (Iatcu et al., 2022). Hong et al. observed a significant increase in the abundance of bacteria such as *Clostridia, Lachnospirales* and *Oscillospirales* in the gut microbiome of DN mice, while the abundance of *Clostridia_UCG-014* decreased significantly. As a protective gut microbiota capable of producing short-chain fatty acids (SCFAs), decreased abundance of *Clostridia_UCG-014* may lead to insufficient production of anti-inflammatory metabolites such as butyrate. This metabolic deficiency could compromise intestinal barrier integrity, potentially facilitating the translocation of endotoxins and uremic toxins [including indoxyl sulfate (IS) and p-cresyl sulfate (pCS)] into systemic circulation. Through the gut-kidney axis, this pathological process may ultimately exacerbate renal injury by allowing increased entry of these toxic compounds into the bloodstream. Concurrently, elevated abundance of pro-inflammatory bacterial taxa including Clostridia, Lachnospiraceae, and Oscillospiraceae may enhance lipopolysaccharide (LPS) production. Upon breaching the intestinal mucosal barrier and entering systemic circulation, LPS activates the TLR4-mediated inflammatory pathway, promoting macrophage polarization toward the M1 phenotype. This process triggers the release of pro-inflammatory mediators such as interleukin-6 (IL-6), NLRP3 inflammasome, and tumor necrosis factor-alpha (TNF-*α*). Sustained activation of these inflammatory components (e.g., NLRP3 inflammasome) could contribute to glomerular podocyte injury and renal fibrogenesis through downstream signaling cascades (Hong et al., 2024). Furthermore, Gogebakan et al. demonstrated that propolis exhibits antioxidant properties, immunomodulatory effects, and gut microbiota-modulating capabilities. Notably, animal studies have revealed that organic selenium compounds, propolis and its natural derivatives, such as caffeic acid phenethyl ester (CAPE), may play significant roles in renal injury repair. These therapeutic effects are primarily mediated through their anti-inflammatory and antioxidant mechanisms, which collectively attenuate oxidative stress and suppress inflammatory cascades in renal tissues (Gogebakan et al., 2012; Talas et al., 2009; Salmas et al., 2017; Talas et al., 2014). In a separate randomized controlled trial (RCT), 30 patients with diabetes were equally allocated to three groups: control, Jingui Renqi Pill (JRP) treatment, and quadruple probiotic consortium (containing Bifidobacterium) treatment, followed by a four-week therapeutic intervention. The study revealed that both the JRP and probiotic groups exhibited significant improvements in 2-h postprandial blood glucose (2hPBG), total cholesterol, and low-density lipoprotein cholesterol (LDL-C) levels compared to the control group. Concurrently, reduced albumin-to-creatinine ratio (ACR) was observed in the intervention groups. These findings suggest that pharmacological modulation of gut microbiota through herbal formulations or probiotic supplementation may help delay the progression of diabetic nephropathy by improving metabolic parameters and renal biomarkers (Zhang et al., 2024). Therefore, intestinal flora plays an important role in the development of DN. Then, exploring the influencing factors of DN pathogenesis from the perspective of intestinal flora may bring new ideas for the prevention and treatment of DN.

In recent years, a considerable number of reviews have conducted in-depth synthesis of specific research issues related to DN, discussing the topic of gut microbiome and DN (Tian et al., 2023; Xu et al., 2024; Ni et al., 2022; Du et al., 2022). Such significant literature growth requires new methods to review and analyze trends within the knowledge domain (Sabe et al., 2022).

Bibliometric analysis is a rigorous method that can be used to explore and analyze large amounts of scientific data. It enables us to unravel the subtle differences in research changes in specific fields, while revealing emerging areas within that domain (Donthu et al., 2021). Through statistical analysis and data visualization, bibliometric analysis can describe the evolution of research on a topic and predict future development trends (Ding and Yang, 2022). At present, this method has been applied to research in various industry fields, such as artificial intelligence, engineering design, environmental studies, and so on (Mariani et al., 2023; Zhu et al., 2024; Kut and Pietrucha-Urbanik, 2024). Therefore, this study used Citespace.6.1.R6, VOSviewer1.6.19, and Bibliometrix to analyze the literature on “DN and gut microbiome” published as of December 4, 2024, in order to obtain changes and trends in the hot topics in this field.

## 2 Materials and methods

### 2.1 Data sources

The data source used in this study is WoSCC. In order to cover as many articles as possible, we used terms from the Medical Subject Headings (MeSHs) table in the PubMed database and designed the search equation for this study based on WoSCC’s search rules. Finally, design “Diabetic Nephropathy” and “Gut Microbiome” along with their upstream and downstream vocabulary and synonyms as search terms for retrieval. The search period covers literature published between the start of database construction and December 4, 2024. In Supplementary material, specific search tactics were given.

### 2.2 Data extraction and collection

The literature types included in this study only include research papers and review papers, and the search language is limited to English. This study retrieved a total of 419 records. The next screening was carried out by two researchers at the same time. Through reading the title, abstract, key words and the full text of the literature, it was judged whether the content had a clear correlation with “intestinal flora” and “Diabetic Nephropathy,” and the literature that obviously did not meet the inclusion criteria was de duplicated and excluded. Two researchers retrieved 209 and 240 articles, respectively. Afterwards, the two researchers exchanged results, and the third researcher decided whether to include literature that was difficult to determine. Finally, after excluding 9 Meeting Abstracts, 8 Early Access articles, 2 Corrections and 162 unrelated articles, a total of 238 articles met the inclusion criteria between January 1, 2014 and December 4, 2024. The above screening process was completed within 1 day. The flow chart of the study is shown in Supplementary Figure 1.

### 2.3 Bibliometric analysis

The 238 documents that met the criteria were selected for the study, exported and downloaded as Plain text files, Tab delimited files and BibTeX. We used CiteSpace version 6.1.R6 (Drexel University, Pfizer, America) to extract and organize explosive keywords and journal double images based on their frequency of occurrence, and generated co-occurrence maps. Use VOSviewer version 1.6.18 (Leiden University, Leiden, Netherlands) to identify the output countries, institutions, journals, and keywords, and form a co-occurrence map. Visualize the author using Bibliometrix in RStudio version 4.2.3. Every year, Microsoft Office Excel (v. 2019) is used to manage data and analyze publishing trends for articles and co citations.

Ethical approval was not applicable for the present study.

## 3 Result

### 3.1 Annual publications and citation frequency

**Figure 1:**
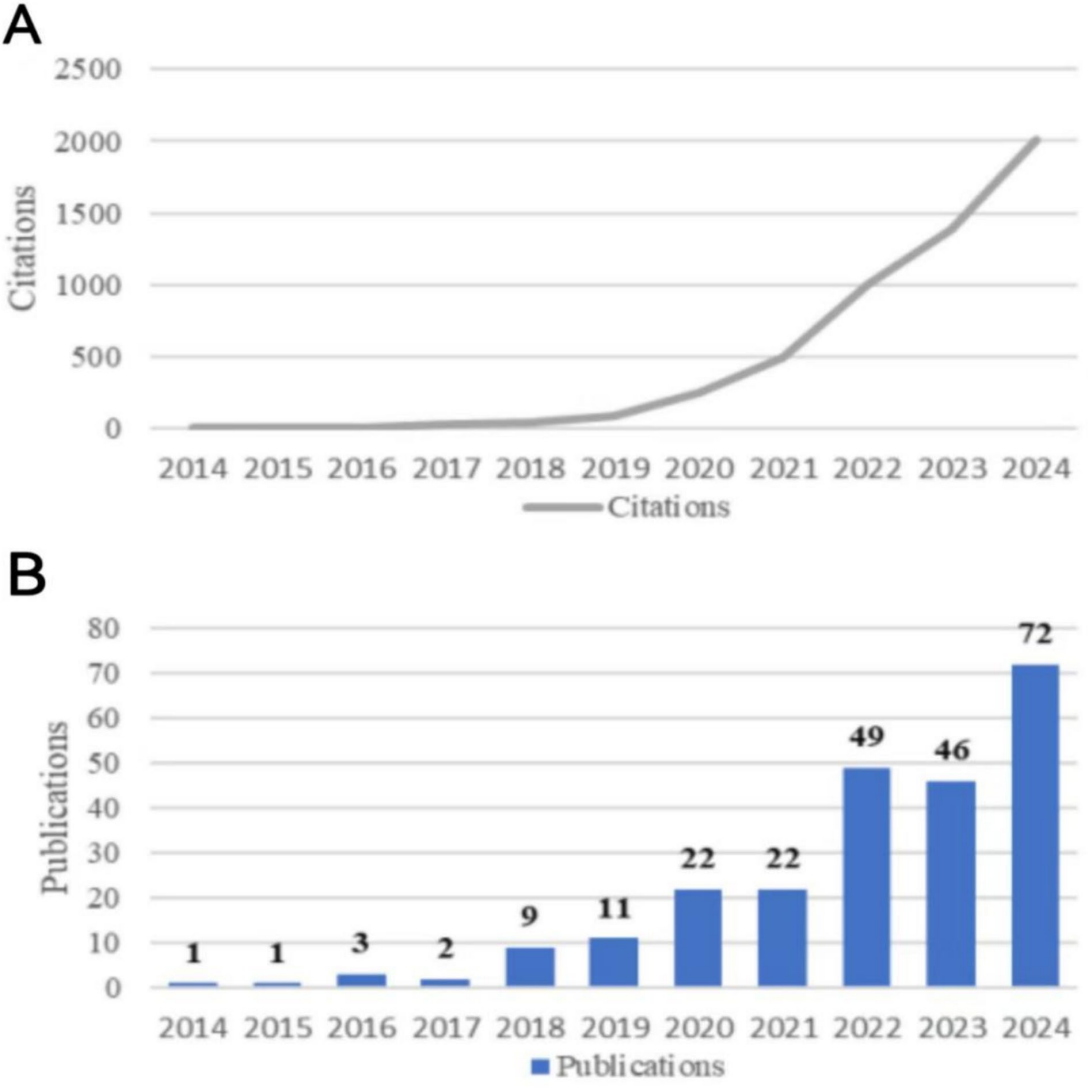
Annual citations for diabetic nephropathy and the gut microbiota (2014 to 2024) **(A)**, and annual publication trends in diabetic nephropathy and the gut microbiota (2014 to 2024) **(B)**.

According to the search formula and screening criteria, a total of 238 articles were included. Among them, there are 64 reviews, 168 articles are open access, Times Cited a total of 5,431 times, with an average citation frequency of 22.82 and an h-index of 40. The trend of annual publication volume and annual citation is shown in Figure 1, showing an overall upward trend. Overall, the annual publication volume in this field can be divided into two stages: (1) 2014–2018: at this time, the annual publication volume did not exceed 10 articles, with slow growth; (2) 2020–2024: The number of publications rapidly increased, slightly decreasing in 2023, with 2024 being the peak year for publication volume.

### 3.2 Journals

Articles related to gut microbiome and DN have been published in 119 journals, such as Supplementary Figure 2, with the top 10 journals listed in Supplementary Table 1. The journal with the highest impact factor is *INTERNATIONAL JOURNAL OF BIOLOGICAL MACROMOLECULES*, and the journal with the highest publication volume is *FRONTIERS IN ENDOCRINOLOGY.* The top 10 journals in terms of publication volume are all Journal Citation Report (JCR) Q1 or Q2 journals, indicating that this field has good prospects and can be published in high-quality journals. In addition, we conducted a journal double image overlay for DN and gut microbiome, as shown in Supplementary Figure 3, to further explore the topic distribution of the journal and the transfer path of disciplinary knowledge. The cited journals are located on the left side of the figure, while the cited journals are located on the right side. There are four main transfer paths in the figure, with labels representing the cited disciplines and paths representing citation relationships. The orange citation pathways indicate that research in *MOLECULAR/BIOLOGY/GENETICS* is frequently cited by journals in *MOLECULAR/BIOLOGY/IMMUNOLOGY* and *VETERINARY/ANIMAL/SCIENCE* domains, while research in *HEALTH/NURSING/MEDICINE* is predominantly cited by journals within the *MOLECULAR/BIOLOGY/IMMUNOLOGY* category. The green citation pathway reveals that studies published in *MOLECULAR/BIOLOGY/GENETICS* journals demonstrate significant cross-disciplinary impact, being systematically referenced by *MEDICINE/MEDICAL/CLINICAL* journals.

### 3.3 Visualization analysis of authors, countries, and institutions collaboration

**Figure 2:**
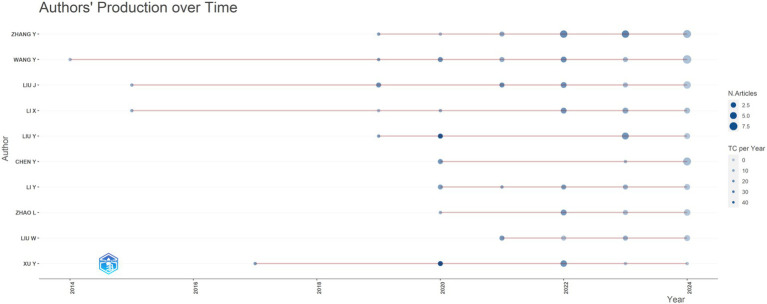
The quantity of articles written by the author fluctuates over time (2014–2024).

A total of 1,603 authors have published articles in this field, the top 10 authors by publication count were depicted in Figure 2. It can be seen that the top 10 authors in terms of publication volume are all from China, with Zhang Yi ranking first in terms of publication volume. Wang, Y initially conducted research on the correlation between DN and gut microbiome.

**Figure 3:**
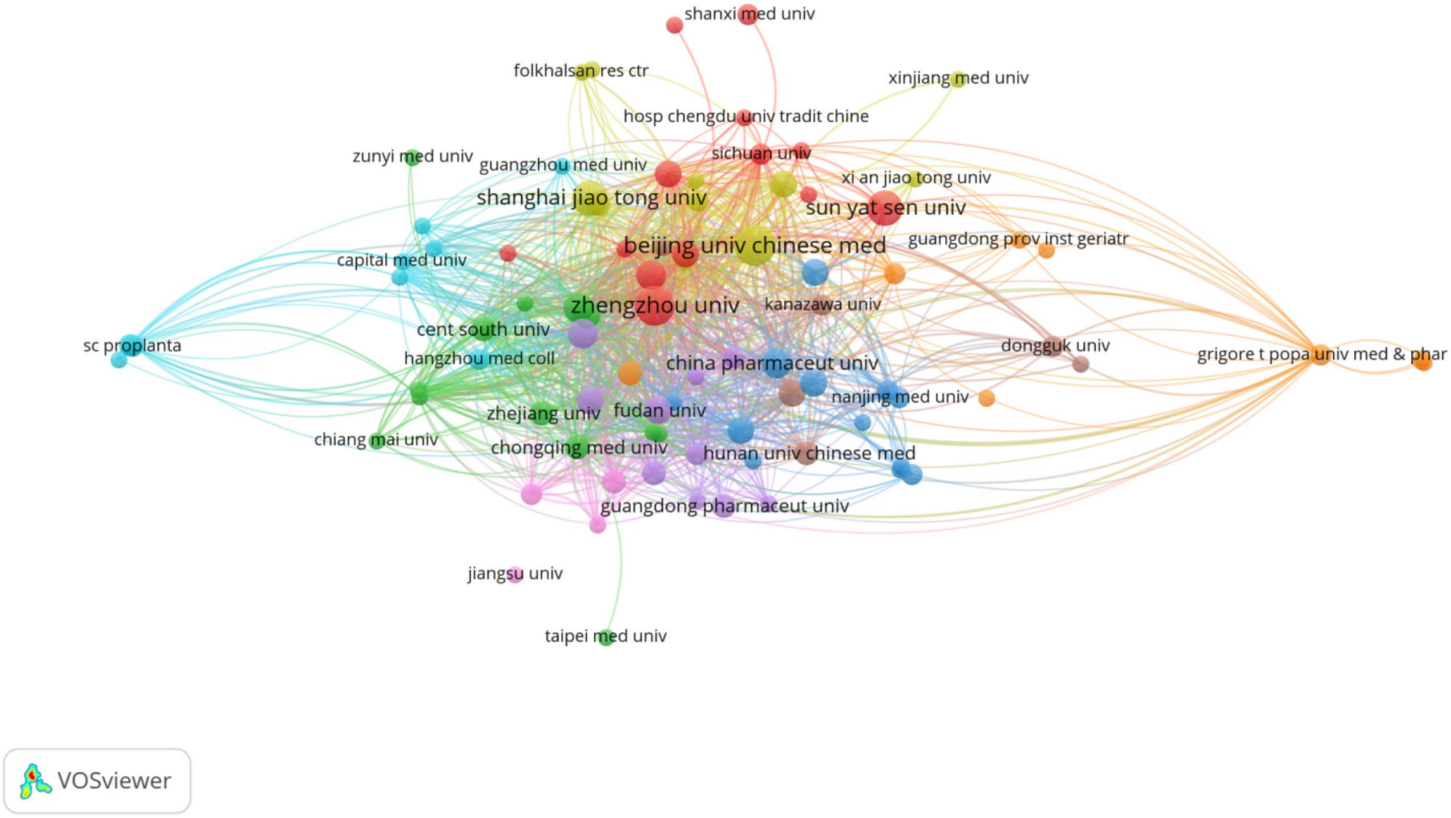
The institution produces visual analyses (2014–2024). Where the size of the nodes represents the volume of publications, and the thickness of the connections represents the level of collaboration.

**Figure 4:**
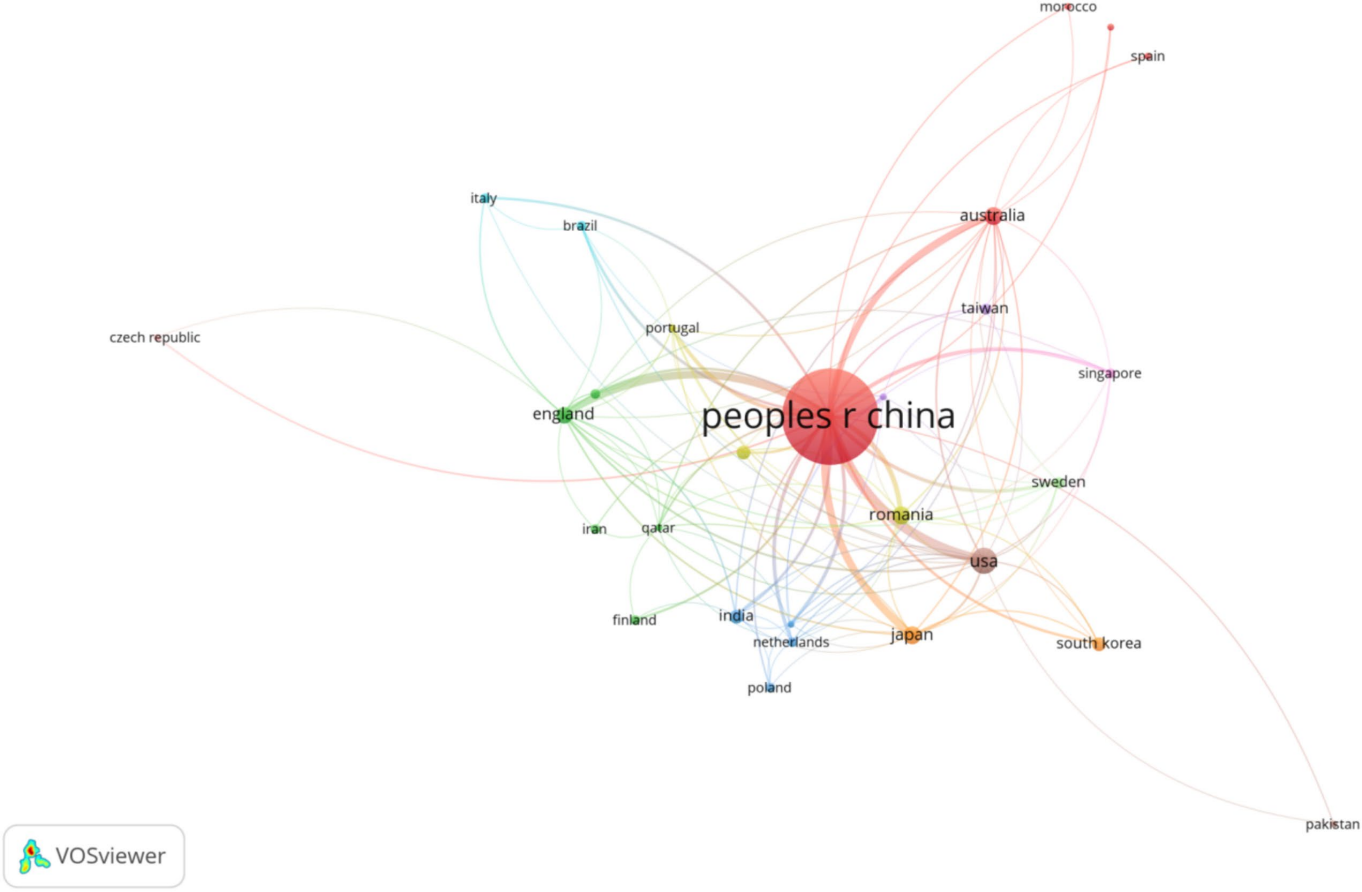
The countries/regions produces visual analyses (2014–2024).

A total of 854 institutions and 52 countries have conducted research and participated in publishing papers in this field, as shown in the visualized graphs in Figures 3, 4. The cooperation between various institutions is frequent, and the top 10 institutions in terms of publication volume are all from China, with Beijing University of Chinese Medicine having the highest publication volume (11 articles). Among countries, China has the highest number of publications (186 articles), followed by the United States (14 articles). The cooperation between countries is relatively close, forming a cooperative relationship with China as the core and various countries cooperating with each other.

### 3.4 Citations

Highly cited articles can help us understand influential research and breakthroughs in this field. Supplementary Table 2 provides detailed information on the top 10 co cited literature. Among the top 10 articles cited, there are a total of 4 reviews and 6 articles. One study explores the potential gut microbiome mechanism of traditional Chinese medicine resveratrol in improving diabetic nephropathy (Cai et al., 2020). One article explores the mechanism of action of phenyl sulfate, a derivative of gut microbiome metabolism, in DN (Kikuchi et al., 2019). There are three articles about short chain fatty acids (SCFAs). Interestingly, two of the articles were published by the same author, who proposed that, SCFAs, Especially butyrate, through G protein coupled receptors 43 (GPR43) mediated oxidative stress and inhibition of NF xB signaling, can improve some T2D induced kidney injury (Huang et al., 2020; Huang et al., 2017). Another article verified that high dietary fiber diet can regulate the intestinal flora of Diabetic Nephropathy mice and increase the production of SCFAs to prevent Diabetic Nephropathy (Li et al., 2020). The regulation of DN by SCFAs through a novel molecular target (the GPR43-*β*-arrestin-2 pathway) was mentioned in both articles (Huang et al., 2020; Li et al., 2020). In addition, only one article used a case–control study to suggest that the ecological imbalance of Gram negative bacteria in the gut microbiome of DN patients may lead to an increase in serum Lipopolysaccharide (LPS) levels in these patients (Salguero et al., 2019).

### 3.5 Keywords and explosive keywords

**Figure 5:**
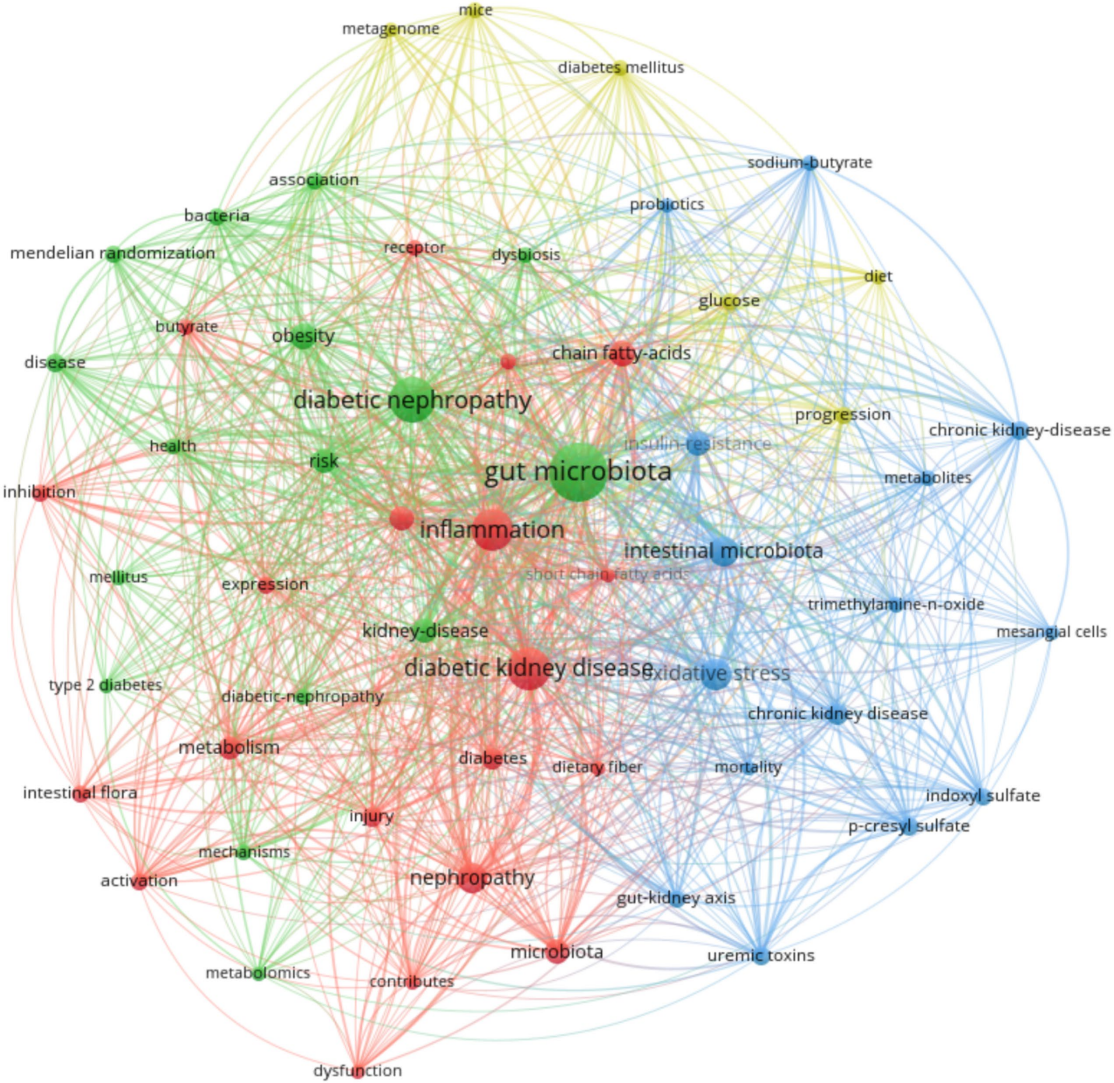
Keyword visual analysis (2014–2024). Node size represents keyword frequency, thickness represents the strength of connection.

Keywords are the core expression of literature and keyword analysis can showcase the research hotspots and frontiers in this field as shown in Figure 5. The top five keywords are gut microbiome (138) diabetic nephropathy (94) diabetic kidney disease (74) inflammation (Koh et al., 2016) and oxidative stress (Joossens et al., 2019). The results of explosive keywords are shown in Supplementary Figure 4. Explosive keywords indicate that a certain keyword appears in a concentrated manner during a certain period of time with high popularity during that time period which can reflect the popularity of the corresponding field. DN and gut microbiome were first related to diet induced obesity appearing in 2014 and continuing until 2018 for a total of 4 years. Currently the focus of attention on explosive hotspots is mainly on uremic toxins short chain fatty acids soy milk and aryl hydrocarbon receptors, which emerged in 2022 and have been ongoing to this day.

## 4 Discussion

### 4.1 General information

#### 4.1.1 Number of publications and journals

This study conducted a bibliometric analysis of the literature related to Diabetic Nephropathy and intestinal flora from the WoS database establishment to December 4, 2024. A total of 238 articles were published in 119 journals. Before 2019, the growth of publications was slow and the field was still in its infancy. In 2024, the number of papers and citations in this field reached the peak, indicating that Diabetic Nephropathy and intestinal flora are becoming research hotspots. The high h-index indicates that this field has high scientific research value. There are still a large number of high IF journals in this field. Among the top 10 journals in terms of publication volume, six belong to the JCRQ1 region, with the highest impact factor being 6.7, which is RENAL Failure. Future researchers can pay attention to cutting-edge journals to obtain the latest research results on DN and gut microbiome.

#### 4.1.2 Countries, institutions, and authors

From the perspective of national publication volume, China has the highest number of publications and the greatest influence. This may be related to the fact that China has the world’s second largest number of diabetes patients, of which about 30–40% of diabetes patients will suffer from Diabetic Nephropathy (Wang et al., 2024; Jia et al., 2025). In addition, the Chinese government is at the forefront of the field as it has proposed the Healthy China Action—Diabetes Prevention and Control Action Implementation Program (2024–2030) literature, which prioritizes the prevention and treatment of diabetes and diabetes-related complications, and as China is a major economic power with sufficient funding for diabetic nephropathy.^1^ The U.S. ranks second in terms of the number of publications, with fewer publications but more citations to the relevant articles. Similarly, the top 10 institutions in terms of publication volume are all from Chinese universities, indicating that conducting this research requires a high academic atmosphere and funding support. Zhang Yi is the first author of the article, and the article with the highest impact factor was published on Gut Microbes. He used a case–control study to explore the role of enterovirus group in type 2 diabetes and DN, and proposed that the characteristics of type 2 diabetes and its complications DN were that the diversity of enterovirus was significantly reduced, multiple virus functions were lost, and virus bacteria correlation was destroyed (Fan G. et al., 2023). Wang, Y has been researching in this field for the longest time. His articles published in Q1 District magazine mainly explore the specific mechanism of relieving DN by Diphenyl deselenide, Zuogui Jiangtang Yisheng, Cordyceps cicadae polysaccharides, etc. through the mouse model of Diabetic Nephropathy (Wang et al., 2024; Yi et al., 2023; Yang et al., 2020).

#### 4.1.3 Citations

From the perspective of highly cited articles, out of the top 10 cited articles, 6 were published in journals in JCRQ1 region. Only one study focused on DN patients, and their results showed that the dysbiosis of the Gram negative phyla (*Proteobacteria, Verrucomicria* and *Fusobacteria*) in the gut microbiome of DN patients is associated with elevated levels of LPS endotoxins in the blood (Cai et al., 2020). Therefore, researchers can explore ways to reduce the relative abundance of these bacteria, such as diet, fecal microbiome transplantation, etc., in order to alleviate inflammation levels in DN patients (Cani et al., 2007). Cohort study can help researchers observe the dynamic changes in gut microbiome in patients undergoing different treatment methods over a long period of time. However, we did not find any published studies on the association between DN and gut microbiome. We look forward to further research from researchers. In the first 10 articles, we can also discover the important role of traditional Chinese medicine in the treatment of diabetic nephropathy. Resveratrol can rebalance the gut microbiome of db/db mice, improve intestinal dysfunction, and alleviate clinical symptoms of diabetic nephropathy (Cai et al., 2020). Kikuchi et al. (2019) used untargeted metabolomics to demonstrate that the metabolite phenylsulfate derived from gut microbiome can induce proteinuria and podocyte injury. So, exploring methods to inhibit the production of phenyl sulfate can provide new ideas for developing drugs to prevent DN.

### 4.2 Research hotspots

Keywords can reflect the evolution and hot topics in this field. Analyzing the keyword explosion graph and keyword co-occurrence graph can explore current and future research hotspots and trends. During this process, we identified several emerging research trends in the field. These trends include “Inflammation,” “Oxidative stress,” “uremic toxin,” “Aryl hydrocarbon receptors,” “SCFAs,” and “soy milk.” The following summarizes these areas.

#### 4.2.1 Uremic toxin and aryl hydrocarbon receptor

Urinary toxin is a metabolite produced by daily food intake, which is actively transported into urine through glomerular filtration or proximal renal epithelial cells (Ravid et al., 2021). The European Uremic Toxin Work Group (EUTox) categorizes uremic toxins into three types based on their physicochemical properties: water-soluble small molecules (<500 Da), intermediate molecules (>500 Da), and protein bound uremic toxin (Vanholder et al., 2003). The disrupted gut microbiome of DN patients can promote the production of certain protein end products, ultimately leading to the production of uremic toxins such as p-cresol sulfate (PCS), indole sulfate (1S), phenyl sulfate (PS), and trimethylamine n-oxide (TMAO) in the liver (Kikuchi et al., 2019; Gryp et al., 2020). Joosens et al.’s study showed that Akkermansia, Bacteroides, Blautia, Enterococcus, Dialeste, and Ruminococcus are associated with an increase in uremic toxins (Joossens et al., 2019). Aryl hydrocarbon receptor (AhR) is an important receptor for uremic toxins. Recent studies have shown that the activation of AhR caused by uremic toxin accumulation may be related to DN (Liu et al., 2021). And indophenol sulfate (IS) is a newly discovered AhR ligand (Schroeder et al., 2010). When AhR binds to IS, it is activated and translocated to the nucleus, leading to transcription of target genes and causing renal inflammation and fibrosis (Wakamatsu et al., 2018). So, inhibiting the binding of AhR ligands to IS can provide new ideas for preventing DN. The study of Zhao et al. (2020) showed that oral administration of Tangshen Formula can prevent intestinal dysbiosis in DN rats, downregulate AhR, and inhibit gut derived IS.

#### 4.2.2 Inflammation and oxidative stress

In the etiology of DN, the roles of “inflammation” and “oxidative stress” are crucial. Under physiological and pathological conditions, the complex interaction between oxidative stress and inflammation forms a “vicious cycle” of mutual feedback (Wu et al., 2020). After inflammation activation, immune system cells secrete cytokines and pro-inflammatory chemokines, stimulating the production of reactive oxygen species (ROS) and reducing antioxidant factor (Tanase et al., 2022). The excessive production of ROS can lead to inflammation, fibrosis, endothelial dysfunction, and subsequent ESRD in the kidneys (Simon et al., 2000). In diabetes patients with intestinal dysbacteriosis, the intestinal barrier becomes more permeable, leading to the leakage of lipopolysaccharide (LPS) into the blood, further leading to inflammation and aggravating the development of DN (Wu et al., 2023). At present, some natural extracts such as Punicalagin and Resveratrol have been found to have effects in reducing oxidative stress, anti-inflammatory, and regulating gut microbiome in diabetic nephropathy (Du et al., 2022). We found that the correlation between promoting the production of SCFAs by bacteria and alleviating DN anti-inflammatory and oxidative stress inhibition is gradually being discovered by researchers.

#### 4.2.3 SCFAs

The decrease of SCFAs level in the intestinal tract caused by intestinal ecological imbalance in diabetes patients is closely related to the occurrence of DN (Zhao et al., 2018). SCFAs are the main products of dietary fiber fermented by gut microbiome, with the majority being acetate, propionate, and butyrate (Koh et al., 2016). Acetate and butyrate can inhibit oxidative stress, NF kB pathway, and inflammatory response of mesangial cells; Propionate can improve the renal injury induced by some type 2 diabetes by inhibiting the expression of inflammatory indicators such as IL-2, I-17, and IL-6 (Huang et al., 2020). *Faecalibacterium, Fusicatenibacter, Lachnospiraceae_NK4A136_group, Roseburia, Ruminococcus_1 and Coprococcus_3* It has been confirmed that it can produce butyrate salts (van den Munckhof et al., 2018; Han et al., 2022; Belteky et al., 2023; Vijay et al., 2021). So, researchers can explore whether increasing the relative abundance of SCFAs producing bacteria in DN patients can improve kidney damage in the long term. At present, research on SCFAs focuses on their role in regulating energy metabolism, inhibiting inflammation and oxidative stress, regulating immune responses, and anti-fibrotic effects *in vivo* (Huang et al., 2020; Hu et al., 2018; Kim et al., 2013). It is worth noting that inhibiting renal fibrosis is an important method to delay the progression of end-stage renal disease (ESRD) in diabetic nephropathy. A study has found that a bacterium that produces SCFAs, *Ruminococcus gnavus*, is negatively correlated with vascular calcification, which is an independent risk factor for cardiovascular death in ESRD patients (Bao et al., 2023). So its potential mechanism and whether it can be used as a new therapeutic target to treat or delay ESRD require further exploration by researchers.

The current research direction is to use drugs to restore gut microbiome, improve uremic toxins and SCFAs levels, and alleviate diabetic nephropathy. In addition to conventional drugs for treating DN, traditional Chinese medicine and its extracts are gradually being discovered by researchers. For example, Achyranthis bidentatae, The Fufang-zhenzhu-tiaozhi formula, Astragalus membranaceus and *Salvia miltiorrhiza* Chinese herbal extracts can regulate the gut microbiome and promote the production of beneficial SCFAs (Si et al., 2024; Lan et al., 2023; Shen et al., 2023). Overall, these studies indicate that traditional Chinese medicine has potential application value in preventing diabetic nephropathy. However, we did not find any specific cohorts or case–control studies on the use of traditional Chinese medicine in clinical patients.

#### 4.2.4 Soy milk

Diet is also an important factor affecting the structure and function of gut microbiome. Probiotic soybean milk can alleviate glomerular injury and tubulointerstitial lesions by reducing inflammation, proinflammatory cytokines and oxidative stress (Paul et al., 2022). In a randomized double-blind placebo-controlled trial, 44 patients were randomly assigned to receive 200 mL/d soybean milk containing *Lactobacillus plantarum A7*. After 8 weeks, renal function indicators improved (proteinuria, serum creatinine, and glomerular filtration rate) (Abbasi et al., 2018). Babashahi et al. (2020) proved through an experimental study that the mixed consumption of probiotic soybean milk and *Cuminum cyminum* will have a positive effect on reducing FBS and blood lipids in STZ-NA diabetes rats. This discovery needs to be investigated and practiced in human experiments. At present, there are also reports on the beneficial effects of soy milk in preventing DN. Jheng et al. (2017) found that Low fat soy mixture powder inhibited myofibroblast differentiation, renal injury and renal macrophage infiltration, thereby delaying the progression of DN in diabetes patients. However, we did not find any longitudinal studies that further consider individual differences among patients. There are also few reports on adding other probiotics to soy milk. In addition, whether these soy milk can be used as adjuvant therapy in clinical practice still requires the release of relevant reports.

### 4.3 Research trends

At present, the diagnosis of early DN in clinical practice is relatively difficult, as it is detected in stage III or IV, which greatly increases the medical burden (Chen et al., 2016). SCFAs are therapeutic targets, so future research can start from the metabolites of gut microbiome to search for biomarkers for early DN, achieving early detection, diagnosis, and treatment, slowing down the progression of DN, and improving patients’ life expectancy and quality of life. The animal model of Li et al. (2023) shows that acetate can prevent DN. However, whether the results of animal models can be extrapolated to humans and the role of SCFAs and gut microbiome metabolites in the future are still worth exploring.

At present, the mechanism of action of metabolites in gut microbiome is not clear. In the future, multi omics studies can be used, and proteomics can help reveal the selective mechanism of inter species competition in gut microbiome for dietary fiber, thus further advancing research on dietary intervention for diabetic nephropathy. And the role of gut virus groups in DN is rarely explored. Studies have shown that transplanting fecal virus groups can normalize glucose tolerance in mice (Zeng et al., 2024). Therefore, exploring the role of viral communities in DN may become a new therapeutic direction in the future. In addition, most of the current research on the correlation between DN and gut microbiome has been conducted in animal models, with few clinical studies. Due to the differences in gut microbiome among different species, the correlation between gut microbiome and DN should be further studied and validated in humans.

Although treatment methods related to microbial regulation, such as traditional Chinese medicine or soy milk, have shown potential to alleviate the deterioration of kidney health. However, in future clinical trials, further research should be conducted on the most promising nutritional supplements. *In vitro* and *in vivo* studies can help us understand the potential mechanisms of action of these formulations, thereby improving the selection, dosage, and administration standards for future clinical trial studies. However, the overall clinical data of soy milk have not yet supported their consistent use at the bedside, and there have been no reports on the validation of the effectiveness of traditional Chinese medicine in long-term use in clinical patients. Future clinical trials will help us better understand their therapeutic potential.

## 5 Conclusion

As of December 4, 2024, this study evaluated 238 articles on DN and gut microbiome using CiteSpace, VOSviewer, and Bibliometrix. Our analysis reveals an emerging field with contributions from 52 countries, 854 institutions, and 1,603 authors. Inflammation, oxidative stress, and the production of urinary toxins in DN patients are the directions for researchers to explore the mechanisms related to DN patients and gut microbiome. AhR, SCFAs, Traditional Chinese medicine and soy milk provide researchers with treatment ideas for diabetic nephropathy. The application of multi omics analysis and gut virome requires researchers’ attempts. However, current research on DN and gut microbiome is mainly in the laboratory stage. Exploring the specific mechanisms and therapeutic effects of DN and gut microbiome requires cohort studies and clinical trials for validation. In summary, the exploration of the mechanism of action and therapeutic or adjuvant therapeutic targets of the gut microbiome and its metabolites in DN patients may become a research hotspot in the future direction of DN and gut microbiome.

## 6 Limitations

This study only used the WoSCC database. Due to the challenge of merging formats of different databases in bibliometrics. Moreover, bibliometrics typically relies on the WoS database, which is the most reliable citation index in global scientific and academic research. In addition, the quality of non-core databases is generally lower than WoS, so our data results are reliable. On the other hand, CiteSpace analysis depends on the selection of keywords, which may vary from researcher to researcher. This subjectivity in keyword selection may affect the final results of the analysis and may lead to the omission of some important areas of research.

## Data Availability

The original contributions presented in the study are included in the article/Supplementary material, further inquiries can be directed to the corresponding author.

## Footnotes

^1^ https://www.gov.cn/zhengce/zhengceku/202407/content_6965000.htm
