# Supplementary material for: A bibliometric study of global trends in diabetic nephropathy and intestinal flora research
Source: Front Microbiol. 2025 May 21;16:1577703. doi: 10.3389/fmicb.2025.1577703 (PMC12133887; doi:10.3389/fmicb.2025.1577703)
Supplement: Supplementary file 1 [file Supplementary_file_1.doc]

Supplementary Material 1

# **Diabetic Nephropathy and intestinal flora term**

(Diabetic Nephropathy OR Diabetic Kidney Disease OR Diabetic Kidney)AND(Gastrointestinal Microbiome OR Gastrointestinal Microbiomes OR Microbiome, Gastrointestinal OR Gut Microbiome OR Gut Microbiomes OR Microbiome, Gut OR Gut Microflora OR Microflora, Gut OR Gut Microbiota OR Gut Microbiotas OR Microbiota, Gut OR Gastrointestinal Flora OR Flora, Gastrointestinal OR Gut Flora OR Flora, Gut OR Gastrointestinal Microbiota OR Gastrointestinal Microbiotas OR Microbiota, Gastrointestinal OR Gastrointestinal Microbial Community OR Gastrointestinal Microbial Communities OR Microbial Community, Gastrointestinal OR Gastrointestinal Microflora OR Microflora, Gastrointestinal OR Gastric Microbiome OR Gastric Microbiomes OR Microbiome, Gastric OR Intestinal Microbiome OR Intestinal Microbiomes OR Microbiome, Intestinal OR Intestinal Microbiota OR Intestinal Microbiotas OR Microbiota, Intestinal OR Intestinal Microflora OR Microflora, Intestinal OR Intestinal Flora OR Flora, Intestinal OR Enteric Bacteria OR Bacteria, Enteric)
