# Supplementary material for: A bibliometric study of global trends in diabetic nephropathy and intestinal flora research
Source: Front Microbiol. 2025 May 21;16:1577703. doi: 10.3389/fmicb.2025.1577703 (PMC12133887; doi:10.3389/fmicb.2025.1577703)
Supplement: Supplementary file 2 [file Supplementary_file_2.doc]

Supplementary Material 2

**supplementary figure 1** **Retrieval process flowchart for the research** 2

**supplementary figure 2 Network visualization map of journal co-citation analysis generated by VOSviewer.**. 3

**supplementary figure 3 The dual-map overlay of journals in Diabetic Nephropathy and intestinal flora** 4

**supplementary figure 4 Keywords outbreak** 5

**supplementary Table 1 Top 10 most productive journals** 6

**supplementary Table 2 Top 10 highly cited literature** 7


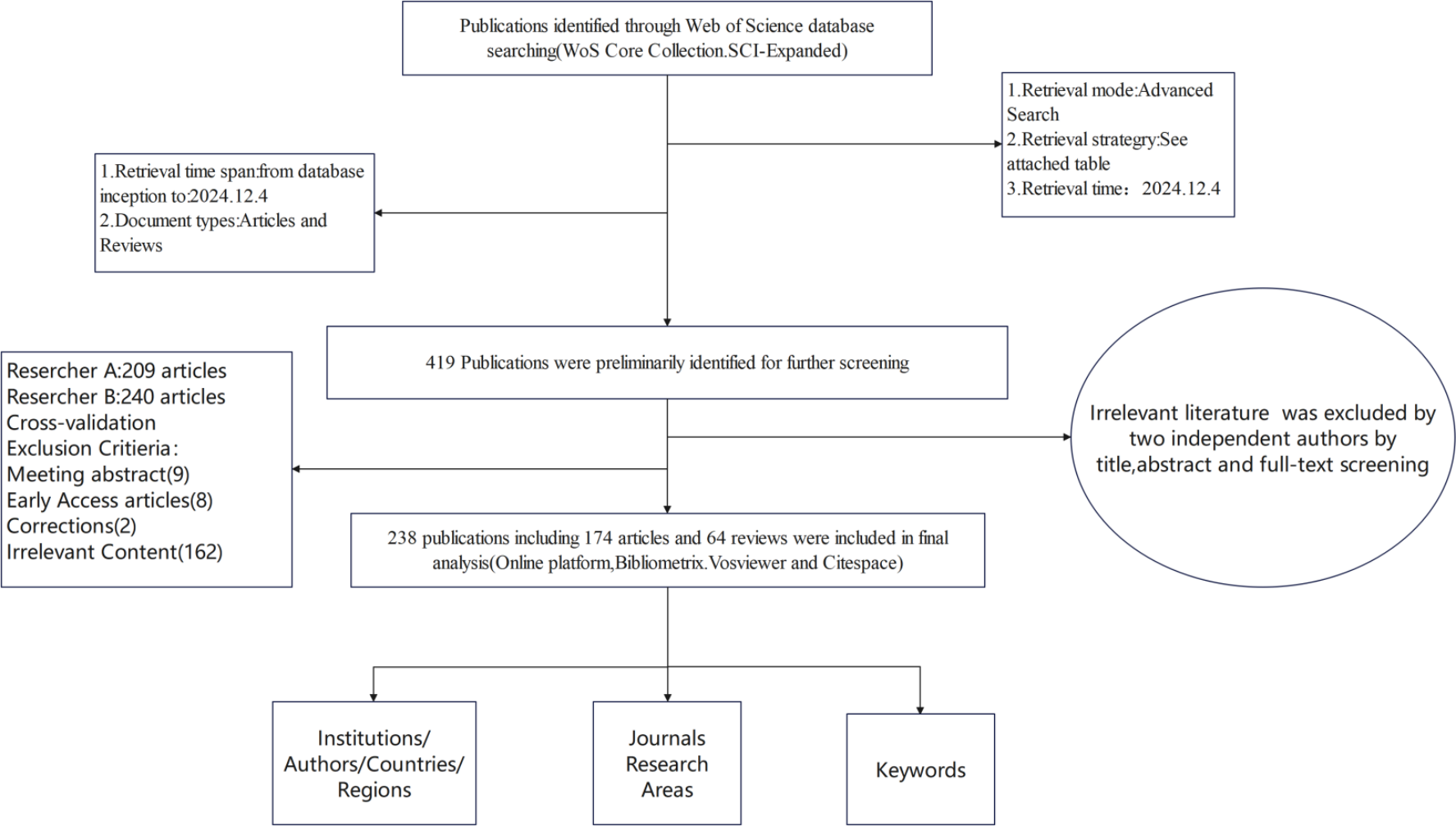


**Supplementary Figure 1：**Retrieval process flowchart for the research.


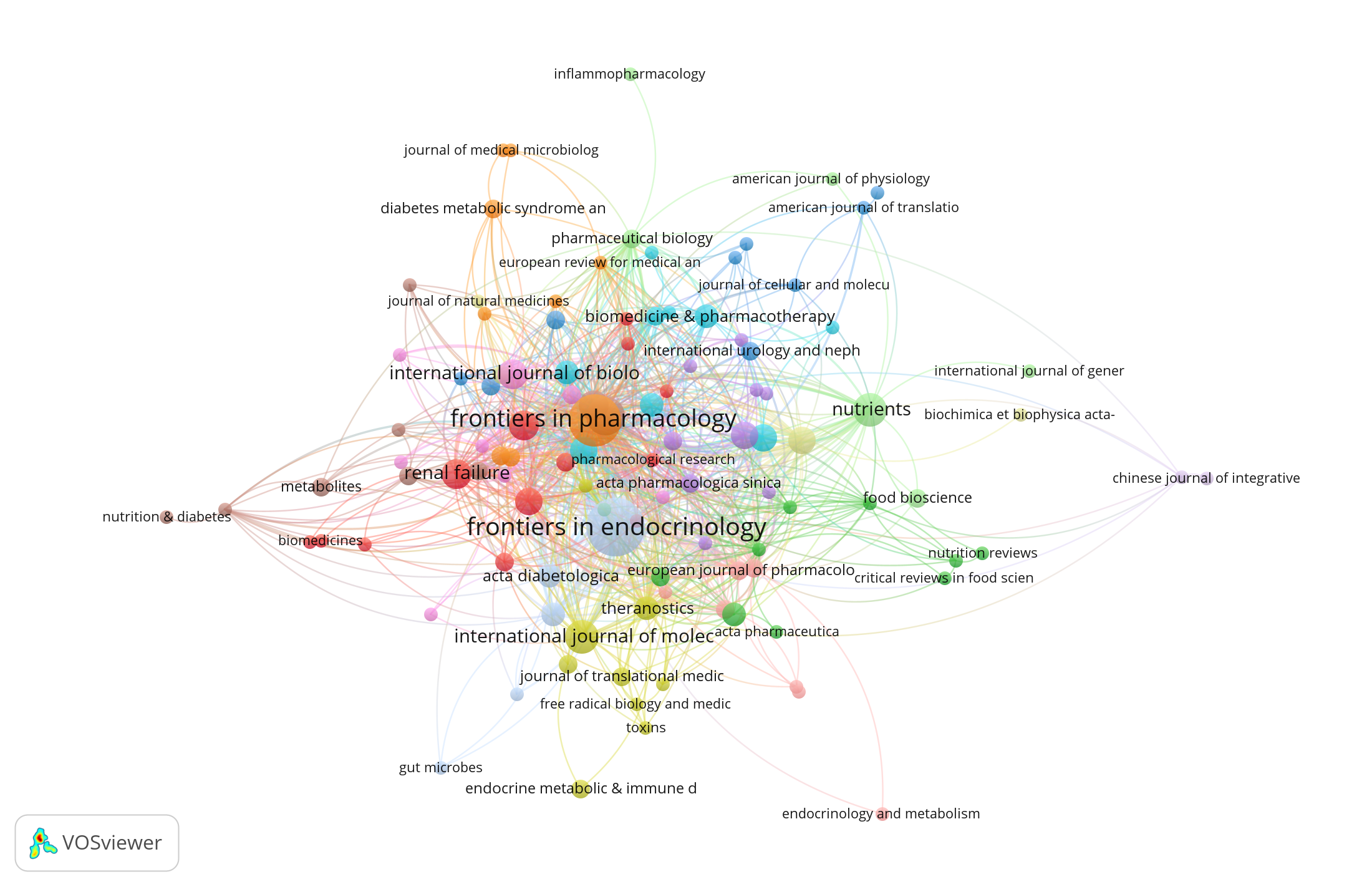


###### Supplementary Figure 2 : Network visualization map of journal co-citation analysis generated by VOSviewer.(2014-2024)


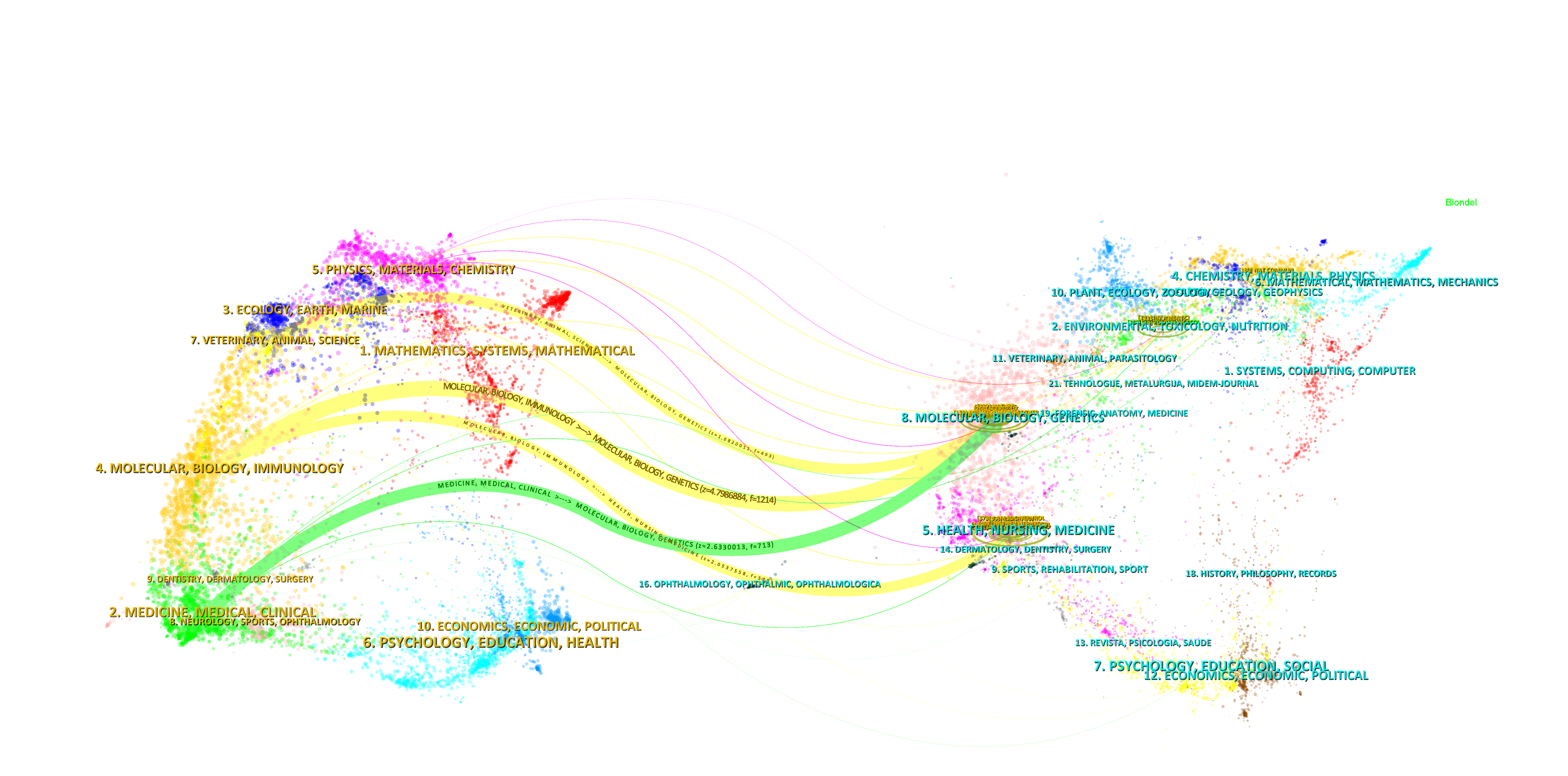
**Supplementary Figure 3：The dual-map overlay of journals in Diabetic Nephropathy and intestinal flora**

**Note:** A dual-map overlay of journals shows the distribution of topics. The citing journals are on the left, and the cited journals are on the right. The labels represent the disciplines covered by the journals, and the colored path represents the citation relationship.(2014-2024)


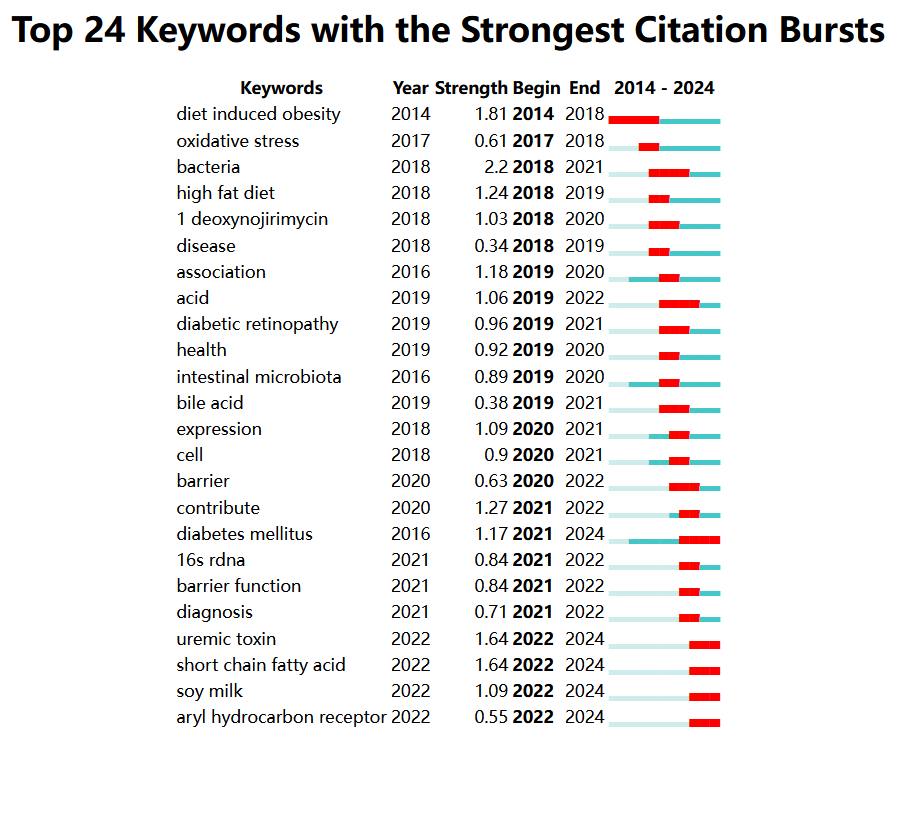


**Supplementary Figure 4：**Keywords outbreak

**Note:**When the end is marked in red, it indicates that the keyword may become a focus in the future.

###### Supplementary Table 1：The top 10 most productive journals.

| Ranking | Journal | Output | % of 90 | IF  2023 | JCR 2023 |
| --- | --- | --- | --- | --- | --- |
| 1 | FRONTIERS IN ENDOCRINOLOGY | 18 | 7.563 | 3.9 | Q2 |
| 2 | FRONTIERS IN PHARMACOLOGY | 15 | 6.303 | 4.4 | Q1 |
| 3 | FRONTIERS IN MICROBIOLOGY | 8 | 3.361 | 4.0 | Q2 |
| 4 | INTERNATIONAL JOURNAL OF MOLECULAR SCIENCES | 6 | 2.521 | 4.9 | Q1 |
| 5 | NUTRIENTS | 6 | 2.521 | 4.8 | Q1 |
| 6 | INTERNATIONAL JOURNAL OF BIOLOGICAL MACROMOLECULES | 5 | 2.101 | 7.7 | Q1 |
| 7 | JOURNAL OF DIABETES RESEARCH | 5 | 2.101 | 3.6 | Q2 |
| 8 | PHYTOMEDICINE | 5 | 2.101 | 6.7 | Q1 |
| 9 | RENAL FAILURE | 5 | 2.101 | 3.1 | Q1 |
| 10 | FOOD FUNCTION | 4 | 1.681 | 5.1 | Q1 |

###### Supplementary Table 2: The top 10 highly cited literature.

| **Title** | **Journals** | **First author** | **Year** | **Citations** | **Citations**/**Year** |
| --- | --- | --- | --- | --- | --- |
| [Role of the gut microbiota in type 2 diabetes and related diseases](https://webofscience.clarivate.cn/wos/woscc/full-record/WOS:000632688100007) | METABOLISM-CLINICAL AND EXPERIMENTAL | Yang | 2021 | 197 | 49.25 |
| [Dietary Fiber Protects against Diabetic Nephropathy through Short-Chain Fatty Acid?Mediated Activation of G Protein?Coupled Receptors GPR43 and GPR109A](https://webofscience.clarivate.cn/wos/woscc/full-record/WOS:000555522300016) | JOURNAL OF THE AMERICAN SOCIETY OF NEPHROLOGY | Li | 2020 | 196 | 39.20 |
| [Gut microbiome-derived phenyl sulfate contributes to albuminuria in diabetic kidney disease](https://webofscience.clarivate.cn/wos/woscc/full-record/WOS:000465200000016) | NATURE COMMUNICATIONS | [Kikuchi](https://webofscience.clarivate.cn/wos/author/record/9820386) | 2019 | 192 | 32.00 |
| [Gut Microbiota and Complications of Type-2 Diabetes](https://webofscience.clarivate.cn/wos/woscc/full-record/WOS:000741821400001) | NUTRIENTS | [Iatcu](https://webofscience.clarivate.cn/wos/author/record/2302933) | 2022 | 169 | 56.33 |
| Dysbiosis of Gram-negative gut microbiota and the associated serum lipopolysaccharide exacerbates inflammation in type 2 diabetic patients with chronic kidney disease | EXPERIMENTAL AND THERAPEUTIC MEDICINE | [Salguero](https://webofscience.clarivate.cn/wos/author/record/14185296) | 2019 | 204 | 29.10 |
| Short-Chain Fatty Acids Ameliorate Diabetic Nephropathy via GPR43-Mediated Inhibition of Oxidative Stress and NF-κB Signaling | OXIDATIVE MEDICINE AND CELLULAR LONGEVITY | Huang | 2020 | 160 | 26.67 |
| Resveratrol Modulates the Gut Microbiota and Inflammation to Protect Against Diabetic Nephropathy in Mice | FRONTIERS IN PHARMACOLOGY | Cai | 2020 | 153 | 25.50 |
| Short-Chain Fatty Acids Inhibit Oxidative Stress and Inflammation in Mesangial Cells Induced by High Glucose and Lipopolysaccharide | EXPERIMENTAL AND CLINICAL ENDOCRINOLOGY & DIABETES | Huang | 2017 | 152 | 16.89 |
| [Role of Gut Microbiota on Onset and Progression of Microvascular Complications of Type 2 Diabetes (T2DM)](https://webofscience.clarivate.cn/wos/alldb/full-record/WOS:000602434400001) | NUTRIENTS | Tanase | 2020 | 144 | 24.00 |
| Understanding the gut-kidney axis among biopsy-proven diabetic nephropathy, type 2 diabetes mellitus and healthy controls: an analysis of the gut microbiota composition | ACTA DIABETOLOGICA | Tao | 2019 | 132 | 18.86 |
